# Spousal abuse and psychological repercussions

**DOI:** 10.1192/j.eurpsy.2021.1208

**Published:** 2021-08-13

**Authors:** S. Kolsi, S. Hentati, I. Baati, J. Masmoudi

**Affiliations:** Psychiatry A, CHU Hédi Chaker Sfax, Sfax, Tunisia

## Abstract

**Introduction:**

Spousal abuse (SA) against women, by its frequency and its consequences on the health of the victims, is a public health issue. For this reason, the role of the physician is essential not only in the care of victims but also in the screening of psychological repercussions.

**Objectives:**

To study the risk factors associated with the development of post-traumatic stress disorder(PTSD) in women victims of spousal abuse(SA).

**Methods:**

Descriptive and analytical cross-sectional study conducted at the National Health Fund of Sfax(CNSS)on 110 women who consulted during the months of October and November2019.The sociodemographic and clinical characteristics of the consultants were collected using a pre established form.We used a 10-item scale,the “Women’s Experience with Battering Scale” (WEBS),to screen women for SA.PTSD was assessed using a PCLS scale(17 items).

**Results:**

(SA)was estimated at 57.3% in our population.The average WEBS score among abused women was 30.92.The prevalence of PTSD in abused women was 63.5% and the average PCLS score was 48.8.The somatic(p=0.049)and psychiatric(p=0.005)histories in the women who had experienced(SA) were related to the development of PTSD.The PCLS score was significantly associated with the WEBS score(p<.0001 and r=.76).The type of violence experienced (physical, psychological, sexual and material) was correlated with the development of PTSD;(p were respectively: <.0001; <.001; 0.02; <.0001).Similarly, repeated violence was strongly related to it(p<0.001).

**Conclusions:**

It seems clear that the(SA)experienced by the women had a psychological impact through the development of PTSD.In addition,several other risk factors inherent to women can be incriminated in this disorder for which systematic screening remains a necessity in order to allow an apdate care.

**Conflict of interest:**

No significant relationships.

